# Intraspecific difference of *Latilactobacillus sakei* in inflammatory bowel diseases: Insights into potential mechanisms through comparative genomics and metabolomics analyses

**DOI:** 10.1002/imt2.136

**Published:** 2023-09-25

**Authors:** Yaru Liu, Hui Duan, Ying Chen, Chengcheng Zhang, Jianxin Zhao, Arjan Narbad, Fengwei Tian, Qixiao Zhai, Leilei Yu, Wei Chen

**Affiliations:** ^1^ State Key Laboratory of Food Science and Resources Jiangnan University Wuxi China; ^2^ School of Food Science and Technology Jiangnan University Wuxi China; ^3^ National Engineering Research Center for Functional Food Jiangnan University Wuxi China; ^4^ International Joint Research Laboratory for Probiotics Jiangnan University Wuxi China; ^5^ Gut Health and Microbiome Institute Strategic Programme Quadram Institute Bioscience Norwich UK

**Keywords:** comparative genomics, gut microbiota, inflammatory bowel disease, *Latilactobacillus sakei*, metabolomics, probiotic

## Abstract

Inflammatory bowel diseases (IBDs) are chronic inflammatory diseases of the gastrointestinal tract that have become a global health burden. Studies have revealed that *Latilactobacillus sakei* can effectively alleviate various immune diseases, including colitis, rheumatoid arthritis, and metabolic disorders. Here, we obtained 72 strains of *L. sakei* from 120 fermentation and fecal samples across China. In total, 16 strains from different sources were initially screened in an in vitro Caco‐2 model induced by dextran sulfate sodium. Subsequently, six strains (four exhibiting effectiveness and two exhibiting ineffectiveness) were selected for further validation in an in vivo colitis mouse model. The results demonstrated that *L. sakei* strains exhibited varying degrees of amelioration of the colitis disease process. Notably, *L. sakei* CCFM1267, the most effective strain, significantly restored colon length and tight‐junction protein expression, and reduced the levels of cytokines and associated inflammatory enzymes. Moreover, *L. sakei* CCFM1267 upregulated the abundance of *Enterorhabdus*, *Alloprevotella*, and *Roseburia*, leading to increased levels of acetic acid and propionic acid. Conversely, the other four strains (*L. sakei* QJSSZ1L4, QJSSZ4L10, QGZZYRHMT1L6, and QGZZYRHMT2L6) only exhibited a partial remission effect, while *L. sakei* QJSNT1L10 displayed minimal impact. Therefore, *L. sakei* CCFM1267 and QJSNT1L10 were selected for further exploration of the mechanisms underlying their differential mitigating effects. Comparative genomics analysis revealed significant variations between the two strains, particularly in genes associated with carbohydrate‐active enzymes, such as the glycoside hydrolase family, which potentially contribute to the diverse profiles of short‐chain fatty acids in vivo. Additionally, metabolome analysis demonstrated that acetylcholine and indole‐3‐acetic acid were the main differentiating metabolites of the two strains. Therefore, the strains of *L. sakei* exhibited varying degrees of effectiveness in alleviating IBD‐related symptoms, and the possible reasons for these variations were attributed to discrepancies in the carbohydrate‐active enzymes and metabolites among the strains.

## Highlights

- Intraspecific variations of *Latilactobacillus sakei* have been observed in their impacts on inflammatory bowel disease in both in vitro and in vivo models.
- Differences in the carbohydrate‐active enzymes of *L. sakei* may exert an indirect influence on the gut microbiota, consequently exerting a more pronounced effect on the short‐chain fatty acids, leading to variations in the degree of remission.
- On the basis of the metabolomic profile of *L. sakei* strains, it was found that acetylcholine and indole‐3‐acetic acid were tentatively identified as key substances that may contribute to the variations in their therapeutic efficacy.

## Graphical abstract

We obtained 72 strains of *Latilactobacillus sakei* from 120 fermentation and fecal samples across China. In total, 16 strains from different sources were initially screened in an in vitro Caco‐2 model. Subsequently, six strains (four exhibiting effectiveness and two exhibiting ineffectiveness) were selected for further validation in an in vivo colitis mouse model. Both in vivo and in vitro findings indicated intraspecific variations of *L. sakei* in their impacts on inflammatory bowel diseases. Notably, differences in the carbohydrate‐active enzymes of *L. sakei* may exert an indirect influence on the gut microbiota, consequently exerting a more pronounced effect on the short‐chain fatty acids, leading to variations in the degree of remission. On the basis of the metabolomic profile of *L. sakei* strains, it was found that acetylcholine and indole‐3‐acetic acid were tentatively identified as key substances that may contribute to the variations in their therapeutic efficacy.

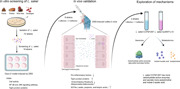

## INTRODUCTION

Inflammatory bowel disease (IBD) is characterized as a chronic inflammatory disorder of the intestinal tract, presenting with symptoms, such as hematochezia, abdominal pain, diarrhea, or indigestion [1]. It is primarily categorized into Crohn's disease (CD) and ulcerative colitis (UC), classifications based on distinct anatomical locations and clinical features [2]. CD causes transmural inflammation with complications including intestinal granulomas, strictures, and fistulas and could influence any area of the intestine (most commonly the terminal ileum and colon), often discontinuously. Conversely, UC triggers inflammation that is localized within the mucosal layers of the rectum and colonic region, which might affect the colon wholly or partly [3, 4, 5]. Epidemiological studies have revealed that IBD affects over 3.5 million people. Since 1990, the incidence of IBD has risen in Asia over the last few decades owing to industrialization and urbanization, changes in lifestyle, and dietary preferences. By 2025, a nation with a high population density, such as China, is estimated to have over 1.5 million cases of IBD if the prevalence rate remains constant at 0.1% [6, 7]. The etiology and pathophysiology of IBD are unknown, however, it is widely postulated that a complex interplay involving the host genome, environmental factors, gut microbiota, intestinal barrier, and immune response contributes to its onset [8, 9, 10]. Thus, gut microbiota dysbiosis is a pivotal pathogenic factor in IBD. Some success has been achieved in IBD treatment; nonetheless, conventional medications do not reduce the overall course of IBD. Despite the extended impact exerted by these established therapeutic agents, a significant proportion of patients continue to exhibit resistance to, or encounter challenges in sustaining treatment with these pharmaceuticals, furthermore, these drugs are accompanied by side effects that encompass infection and malignancy risks [11]. Therefore, using probiotics to alleviate or treat IBD has become a prominent research topic, based on the inescapable role of the gut microbiota.

Some probiotics, including *Bifidobacterium longum*, *Lacticaseibacillus casei*, *Lactobacillus gasseri*, *Bifidobacterium lactate*, and *Lactiplantibacillus plantarum*, are effective in reducing intestinal inflammation in mice with IBD [12, 13, 14], suggesting that studying the contribution of specific gut microbiota to the progression of IBD could unearth effective treatment options. In summarizing the mechanisms by which probiotics in treating IBD, studies have identified three key elements: (i) Inhibiting the growth of pathogenic organisms in the gut. The antimicrobial peptides or other metabolites generated by probiotics possess the capability to inhibit the growth activity of other microorganisms within the gut [15]. For example, short‐chain fatty acids (SCFAs), metabolites of probiotics, can cause specific pH changes in the gut [16], possibly directly inhibiting pathogenic bacterial growth. However, probiotics can compete with enteropathogenic microorganisms for receptors or binding sites on the surface of the intestinal mucosa and inhibit the invasion of epithelium‐attached enterotoxins and enteropathogenic bacteria [17, 18]. (ii) Regulating intestinal immunity. Chronic intestinal inflammation in patients with IBD is caused by aberrant immune system activation. In the intestinal mucosa, dendritic cells are associated with the regulation of the immune system, influencing the differentiation of regulatory T cells (Treg) and helper T cells 17 (Th17), and mucosa‐specific immunoglobulin A production and conversion; activating these processes can potentially cause the generation of proinflammatory cytokines, notably interleukin (IL)‐17 and IL‐23 [19]. In contrast, probiotics affect the immune system in the mucosal layer, stimulating antibody secretion by activating Toll‐like receptors and Th1 differentiation. Probiotics affect the immune system in the mucosal layer, thereby stimulating the secretion of antibodies through the activation of Toll‐like receptors and Th1 differentiation, promoting phagocytosis and the activity of natural killer (NK) cells, inducing T cell apoptosis, and stimulating the secretion of anti‐inflammatory cytokines, like, transforming growth factor‐β (TGF‐β) [20]. However, the secretion of some proinflammatory cytokines (such as TGF‐α and interferon‐γ) is reduced by inhibition [21, 22]. (iii) Improvement of the intestinal barrier. Probiotics improve or restore the intestinal barrier's functionality through multiple mechanisms. These encompass the inhibition of apoptosis among intestinal epithelial cells, the facilitation of tight‐junction (TJ) synthesis, and the attenuation of pathways associated with intestinal barrier permeability. For example, *Lactobacillus reuteri* R2LC enhances the expression of the TJ, Occludin, and ZO‐1, which protect against colitis in mice [23].

*Latilactobacillus sakei*, a human gastrointestinal probiotic widely present in daily food such as sourdough, fermented pickles, and fermented meat products, is often used as a biological initiator and protectant in the food industry owing to its metabolism that produces bacteriocin and flavor compounds [24, 25]. Comparative genomics [26] has revealed the specific genomic profiles of *L. sakei* existing within an extensive accessory of genomes that contain half of the pan‐genome, suggesting that such groups have different environmental preferences. Specific differences between groups and strains can be found, mainly in agmatine and citrate metabolism. The presence of genes encoding pathways for fructose, sucrose, trehalose, and gluconate metabolism across all strains implies a broad adaption to environments rich in plant‐based or sugary constituents. Additionally, proteomics and transcriptomics investigations have been undertaken to elucidate the metabolic profile of *L. sakei* across diverse ecological niches [27, 28]. Probiotic functions also appear to be closely related to their metabolites. Currently, the active metabolites of probiotics that have been extensively studied mainly include exopolysaccharide, bacteriocins, organic acids, SCFAs, and vitamins. These active metabolites have been shown to exhibit properties such as anti‐inflammation, antitumor, antioxidation, immune regulation, and prevention or treatment of various metabolic diseases [29]. Kim et al. [30] and Tsuji et al. [31] conducted metabolomic analyses on *L. sakei* during its fermentation process, illuminating the species' adaptive traits across diverse environments. Notably, *L. sakei* displays a rich spectrum of metabolite production, encompassing extracellular polysaccharides and γ‐aminobutyric acid (GABA), both of which confer notable health benefits to humans. Bajpai et al. [32] observed that the exopolysaccharides generated by *L. sakei* exhibit substantial superoxide dismutase‐like activity and effectively inhibit α‐glucosidase, displaying concentration‐dependent tyrosinase activity and a substantial polyphenol content, indicative of robust antioxidant properties. The GABA produced by *L. sakei* has been linked to enhanced long‐term memory recovery in the murine model [33]. Moreover, when combined with exercise, GABA also demonstrates a significant reduction in weight gain, thus serving as an antiobesity agent [34]. Alterations in the host's processing of signals derived from gut microbiota, alongside shifts in the composition and functionality of the gut microbiota, have strong associations with IBD. The pivotal molecular intermediaries connecting the microbiota and the host consist of metabolites derived from the gut microbiota. Therefore, there has been an exploration of the potential capacity of *L. sakei* to alleviate colitis (Table 1). However, the immunomodulating functions and mechanisms of different isolated sources of this strain have not yet been elucidated. Here, we aimed to screen *L. sakei* from different sources and explore the intraspecific differences of *L. sakei* on IBD, establishing a theoretical basis for further research on *L. sakei* with probiotic functions.

**Table 1:** *Latilactobacillus sakei* with the effect of relieving colitis.

Strain	Animal model	Mechanism	Year of publication	Reference
*L. sakei* CVL‐001	DSS‐induced colitis	Regulate immune response and intestinal integrity via gut microbiota modulation	2023	[35]
*L. sakei* probio65	TNBS‐induced colitis	Reduce proinflammatory cytokines, regulate gut microbiota	2020	[36]
*L. sakei* S1	TNBS‐induced colitis	Inhibit the NF‐κB pathway	2020	[37]
*L. sakei* OK67	HFD‐induced colitis	Inhibit the NF‐κB pathway	2019	[38]
*L. sakei* K040706	DSS‐induced colitis	Inhibit inflammation	2017	[39]
*L. sakei* K17	TNBS‐induced colitis	Upregulate the expression of IL‐10 and TJ, inhibit the NF‐κB pathway	2016	[40]

Abbreviations: DSS, dextran sulfate sodium; HFD, high‐fat diet; IL, interleukin; NF‐κB, nuclear factor kappa B; TJ, tight junction; TNBS, 2,4,6‐trinitrobenzene sulfonic acid.

## RESULTS

### Isolation and screening of *L. sakei*

**Figure 1:**
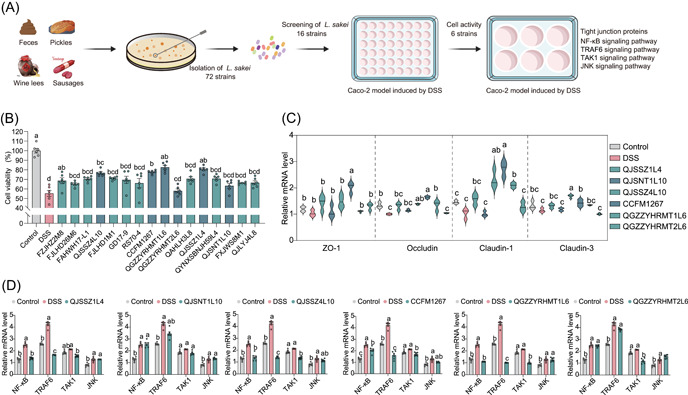
Screening of *Latilactobacillus sakei* in vitro. (A) Flowchart of screening of *L. sakei* in vitro. (B) Impact of *L. sakei* on Caco‐2 cell viability after dextran sulfate sodium (DSS) stimulation. (C) Effects of *L. sakei* on TJ protein in Caco‐2 cells after DSS stimulation. (D) Influence of *L. sakei* on the expression of Caco‐2 cell‐related immune pathway after DSS stimulation. Different lowercase letters as superscripts (a–c) in the graph signify significant differences between groups (*p* < 0.05), determined by Tukey's multiple comparisons test (*n* = 6). JNK, c‐Jun N‐terminal kinase; NF‐κB, nuclear factor kappa B; TAK1, transforming growth factor beta‐activated kinase 1; TJ, tight junction; TRAF6, tumor necrosis factor receptor‐associated factor 6.

A total of 658 strains were isolated and screened from 120 fermentation and feces samples collected across diverse regions within China using a common De Man, Rogosam, and Sharpe (MRS) medium. Totally, 72 strains of *L. sakei* were isolated from nine provinces in China, including Jiangsu, Anhui, Guangdong, Jilin, Zhejiang, Sichuan, Yunnan, Xinjiang, and Guizhou, of which 12, 27, 26, and 7 strains were screened from fecal sources, fermented meat products, fermented vegetables, and fermented wine lees, respectively. Sixteen strains of *L. sakei* were selected from different samples for subsequent experimental studies (Figure 1A).

### Exploring the in vitro immunomodulatory capacity of *L. sakei*

Overall, 16 strains of *L. sakei* from different sources were selected from different samples, and the in vitro immunomodulatory activity of *L. sakei* was preliminarily investigated by the Caco‐2 cell model induced by dextran sulfate sodium (DSS). First, suitable DSS (Figure S1) and bacterial concentrations (Figure S2) were selected. A 3% DSS close to the semilethal safe dose of cells and a bacterial concentration of 10^7^ colony‐forming units (CFU)/mL were used for subsequent experiments. We determined the effect of *L. sakei* on the activity of Caco‐2 cells stimulated by DSS (Figure 1B). The detrimental impact of DSS on the activity of Caco‐2 cells was significantly reduced by *L. sakei* CCFM1267, QGZZYRHMT1L6, QJSSZ1L4, and QJSSZ4L10, which increased the survival rate of Caco‐2 cells to >74%. However, the activity of Caco‐2 cells was 63.23% and 57.94%, which revealed that they did not significantly benefit from the protective effects of *L. sakei* QJSNT1L10 and QGZZYRHMT2L6. The six *L. sakei* strains were selected for follow‐up studies based on the results above. Figure 1D indicates that, except for *L. sakei* QJSNT1L10 and *L. sakei* QGZZYRHMT2L6, the expression level of the nuclear factor kappa B (NF‐κB) gene in Caco‐2 cells was substantially diminished by the four remaining strains. Only *L. sakei* CCFM1267 markedly diminished the expression levels of c‐Jun N‐terminal kinase (JNK) signaling pathway‐related genes. Figure 1C reveals that DSS stimulation downregulates the expression levels of four TJ‐related genes, including Claudin‐1, Claudin‐3, Occludin, and ZO‐1, in Caco‐2 cells—which align with the findings reported by Hwang et al. [41]. However, *L. sakei* restored the expression of TJ protein‐related genes to varying levels in Caco‐2 cells.

### Evaluation of alleviating effect of *L. sakei* on DSS‐induced colitis in mice

**Figure 2:**
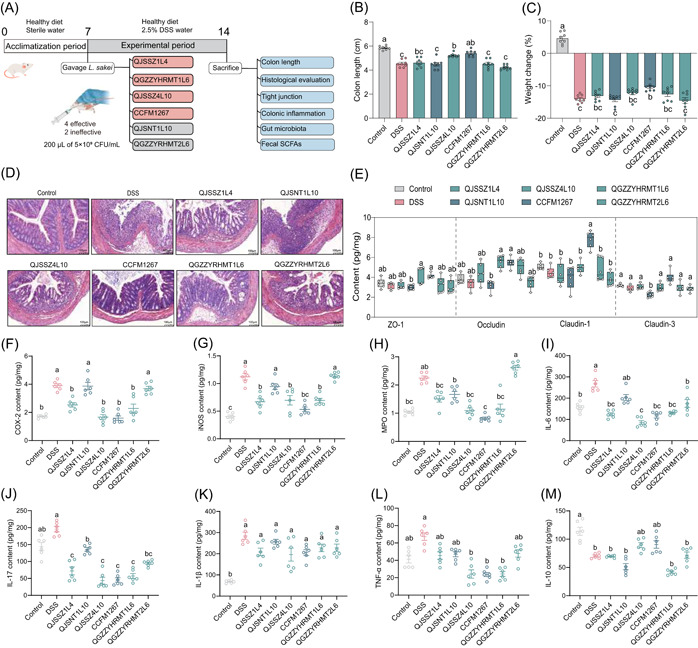
*Latilactobacillus sakei* ameliorates DSS‐induced murine colitis. (A) Flowchart of animal experiment. (B, C) Effects of *L. sakei* on physiological indexes of mice with colitis. (D) Effects of *L. sakei* on the histological morphology of colonics in mice with colitis. (E) Effects of *L. sakei* on colon TJ protein content in colitis mice. (F–H) Effects of *L. sakei* on inflammatory enzyme content in colitis mice. (I–M) Effects of *L. sakei* on cytokine content in colitis mice. Different lowercase letters as superscripts (a–c) in the graph signify significant differences between groups (*p* < 0.05), determined by Tukey's multiple comparisons test (*n* = 8). CFU, colony‐forming units; DSS, dextran sulfate sodium; SCFA, short‐chain fatty acid; TJ, tight junction.

This study was further validated in the mouse model of UC produced by DSS to evaluate the immunomodulatory activity of the aforementioned six strains of *L. sakei* (Figure 2A). The colon length of mice was reduced from 5.8 to 4.5 cm in the control group following DSS molding (Figure 2B and Figure S3). The colon in the DSS group was swollen and bleeding with loose feces, in contrast to that in the control group, which was red with granular feces, as illustrated in their colonic morphology map, which in line with the outcomes reported by Chen et al. [42]. When *L. sakei* QJSSZ4L10 and CCFM1267 were administered, the colon lengths were restored to 5.2 and 5.4 cm, respectively, improving its abnormality. In contrast, the remaining four strains had no protective effect on colon length. During DSS molding, the mice experienced a reduction in body weight, accompanied by loose and bloody stools [43]. This reduction accounted for approximately 15%, which *L. sakei* CCFM1267 significantly alleviated, whereas *L. sakei* QJSNT1L10 and QGZZYRHMT2L6 had no effect (Figure 2C). Figure 2D reveals that the colonic tissue in the control group had a clear structure with a smooth surface of the mucosal layer, regular epithelial cell morphology, and abundant intestinal glands in the lamina propria, without inflammatory cell infiltration. However, DSS caused extensive necrosis in the mucosal layer of the colon and the disappearance of intestinal glands in the lamina propria of mice with moderate neutrophil infiltration, minimal edema in the submucosal layer, and loose connective tissue with a limited extent of inflammatory cell infiltration. After the strain intervention, *L. sakei* CCFM1267 exhibited the best protective effect against colonic injury, with similar colonic tissue status to the control group. Conversely, *L. sakei* QJSNT1L10 aggravated the damage to the colonic tissues of the mice to an extent, with many intestinal glands in the lamina propria of the mice disappearing with necrosis and neutrophil infiltration and a small area of water in the mucosa layer with loose connective tissue arrangement.

Alterations in the content of TJ within mouse colon tissue and inflammation were measured to assess the protectiveness of different *L. sakei* strains on the intestinal barrier. As depicted in Figure 2E, *L. sakei* CCFM1267 significantly upregulated the content of the four types of TJ; in contrast, *L. sake**i*** QJXNT1L10 and QGZZYRHMT2L6 showed no significant restorative effect. *L. sakei* intervention affected the inflammatory factor levels in some ways, particularly *L. sakei* QJSSZ4L10 and CCFM1267, which markedly diminished tumor necrosis factor alpha (TNF‐α), IL‐6, and IL‐17 levels (Figure 2I–L) and increased IL‐10 levels (Figure 2M). Overexpression of cyclooxygenase‐2 (COX‐2), myeloperoxidase (MPO), and inducible nitric oxide synthase (iNOS) in inflammatory cells promotes the IBD process. Additionally, intervention with *L. sakei* CCFM1267 reduced COX‐2, iNOS, and MPO levels to those of the control group, effectively alleviating intestinal inflammation, while *L. sakei* QJSNT1L10 had no significant change (Figure 2F–H).

### Influence of *L. sakei* on gut microbiota and SCFAs in mice

**Figure 3:**
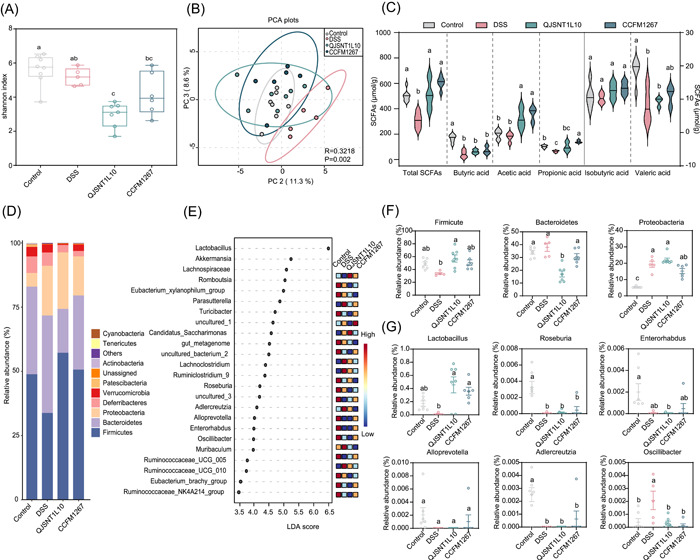
Influence of *Latilactobacillus sakei* on gut microbiota in colitis mice. (A) α‐Diversity of gut microbiota in colitis mice. (B) β‐Diversity of the gut microbiota in colitis mice. (C) Effects of *L. sakei* on SCFAs content in colitis mice. (D, F) Effects of *L. sakei* on the phylum level of the gut microbiota in colitis mice. (E) LEfSe difference of gut microbiota in colitis mice after intervention by *L. sakei*. (G) The relative abundance of different bacterial species after *L. sakei* intervention. Different superscript lowercase letters (a–c) in the graph indicate significant differences between groups (*p* < 0.05) within the row by the Kruskal–Wallis test (Control, *n* = 7; DSS, *n* = 5; CCFM1267, *n* = 6; QJSNT1L10, *n* = 7). DSS, dextran sulfate sodium; LDA, linear discriminant analysis; PC, principal component; PCA, principal component analysis; SCFA, short‐chain fatty acid.

The gut microbiota plays a vital role in human physiological activities, and is involved in pathogen defense, nutrition, metabolism, and immune system regulation [44]. However, gut microbiota may also contribute to UC and colorectal cancer development [45]. In this study, we selected *L. sakei* CCFM1267 and QJSNT1L10—the most and least effective in relieving colitis, respectively—to analyze the regulatory influence of *L. sakei* strains on the flora of mice with colitis. As presented in Figure 3A, *L. sakei* did not significantly modulate the α‐diversity of the gut microbiota. The Shannon index of the *L. sakei* CCFM1267 group was the highest, indicating a higher species richness than that of the other groups. Regarding β‐diversity (Figure 3B), PCA plots [46] revealed some dispersion between the control, DSS, and *L. sakei* intervention groups, indicating the altered structure of the gut microbiota. The phylum level analysis (Figure 3D,F) revealed that *L. sakei* upregulated the DSS‐induced decline in the Firmicutes phylum, accompanied by a downregulation of the abundance of the Bacteroidetes phylum. LEfSe analysis revealed 24 different genera of *Lactobacillus*, *Romboutsia*, *Enterorhabdus*, *Roseburia*, and *Oscillibacter* in the different groups (Figure 3E). *Lactobacillus* spp. in the gut was significantly upregulated after *L. sakei* intervention compared with that of the DSS group (Figure 3G), which may be attributed to the intake of *L. sakei*. *L. sakei* CCFM1267 increased the relative abundance of *Enterorhabdus*, *Roseburia*, and *Alloprevotella* compared with those of *L. sakei* QJSNT1L10 (Figure 3G). *L. sakei* CCFM1267 intervention led to a significant upregulation in *Adlercreutzia* (Figure 3G), a genus that exerts anti‐inflammatory properties through the metabolism of isoflavones [47], compared with that of the DSS group. *Oscillibacter* spp. positively correlated with colitis severity [48]; nevertheless, *L. sakei* CCFM1267 significantly downregulated the relative abundance of this genus (Figure 3G).

SCFAs were also measured in abundance in the feces of mice. As shown in Figure 3C, DSS reduced the total amount SCFAs and decreased the amount of acetic, propionic, and butyric acids compared with those of the control group. When mice were administered *L. sakei*, the levels of each SCFA in the feces exhibited an upregulation. *L. sakei* CCFM1267 revealed a pronounced upregulatory trend of SCFAs compared with *L. sakei* QJSNT1L10. *L. sakei* CCFM1267 produced the most significant upregulation of propionic acid.

### Mechanism of differences in the effect of different bacteria strains on relieving colitis

#### Comparative genomic analysis of differential strains

**Figure 4:**
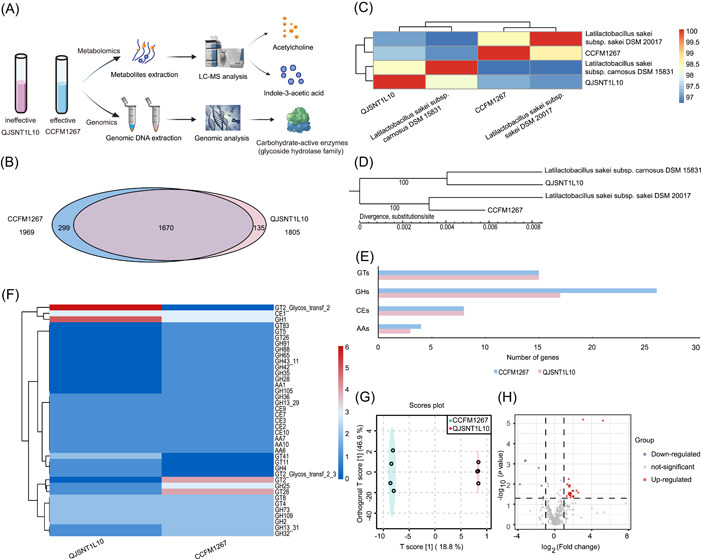
Comparative genomic analysis and metabolome analysis of differential genera. (A) Flowchart of comparative genomic analysis and metabolome analysis of different genera. (B) Analysis of homologous genes of *Latilactobacillus sakei*. (C) Heat map of average nucleotide consistency analysis of *L. sakei*. (D) Phylogenetic analysis of *L. sakei*. (E, F) Carbohydrate‐active enzyme analysis of *L. sakei* from different sources. (G) OPLS‐DA score plot of all metabolites in *L. sakei*. (H) Volcanic map of differential metabolites. AAs, auxiliary activities; CEs, carbohydrate esterases; GHs, glycoside hydrolases; GTs, glycosyl transferases; LC‐MS, liquid chromatography–mass spectrometry; OPLS‐DA, orthogonal partial least squares‐discriminant analysis.

The direct homologous gene analysis (Figure 4B) revealed that both strains of *L. sakei* shared 1670 homologous genes [49]. A significant difference was observed between *L. sakei* QJSNT1L10 and CCFM1267, which have 135 and 299 endemic genes, respectively. Average nucleotide identity (ANI) (Figure 4C) and phylogeny (Figure 4D) revealed that *L. sakei* CCFM1267 and *L. sakei* QJSNT1L10 belonged to two subspecies: *L. sakei* subsp. *sakei* and *L. sakei* subsp. *carnosus*, and the differences in their evolutionary relationships may cause differences in their function‐related genes.

To gain insight into the distribution of functional genes in the genomes of the two *L. sakei* strains, the genomes of both strains were compared using the Clusters of Orthologous Groups (COGs) and the Kyoto Encyclopedia of Genes and Genomes (KEGG) database. Functional genes related to carbohydrate utilization were present in a larger proportion of the genomes of each strain, with some differences (Figures S4 and S5, and Tables S2 and S3). Therefore, the genomes of both *L. sakei* strains were compared with the CAZy database. As shown in Figure 4E, both strains contained the four main families of carbohydrate‐active enzymes, including carbohydrate esterases (CEs), glycoside hydrolases (GHs), glycosyl transferases (GTs), and auxiliary activities (AAs). The AAs, CEs, and GTs families exhibited no significant differences in gene distribution between both strains, whereas, for the GHs family, *L. sakei* CCFM1267 had a larger gene count than those of *L. sakei* QJSNT1L10, with 13 unique genes (Figure 4F).

#### Metabolome analysis of the intraspecific difference

We observed significant differences between the metabolites of both strains using orthogonal partial least squares‐discriminant analysis (OPLS‐DA) (Figure 4G). Using VIP > 1 and *p* < 0.05 as criteria, the differential metabolites between *L. sakei* CCFM1267 and QJSNT1L10 were screened, and the results are depicted in Figure 4H. Overall, 193 metabolites (VIP > 1) and 165 nonsignificantly different metabolites were detected in *L. sakei* CCFM1267 and QJSNT1L10. Significantly different metabolites were further screened using fold change (FC) > 2 or FC < 0.5 as criteria. Thirteen significantly different metabolites were in both *L. sakei* strains (Table 2). Compared with *L. sakei* QJSNT1L10, *L. sakei* CCFM1267 metabolized more acetylcholine and indole‐3‐acetic acid.

**Table 2:** Differential metabolites of *Latilactobacillus sakei* CCFM1267 and *L. sakei* QJSNT1L10.

Differential metabolites	Fold change value	*p* Value
d‐(+)‐Tryptophan	0.0719	0.010272
Cytosine	0.1066	0.000730
4‐Hydroxybutyric acid (GHB)	0.1101	0.000664
Pantothenic acid	0.2970	0.001569
Xanthine	0.3698	0.012159
Cytidine	0.4167	0.043338
3‐Aminophenol	2.4616	0.039459
5′‐*S*‐methyl‐5′‐thioadenosine	2.5757	0.010239
Crotonic acid	2.8615	0.005650
Indole‐3‐acetic acid	2.8703	0.033046
6‐Methylquinoline	3.2949	0.046418
Palmitoyl carnitine	5.1420	0.021209
Acetylcholine	8.4266	0.000006

## DISCUSSION

IBD is a chronic inflammatory disease affecting the gastrointestinal tract, which are predominantly prevalent in North America, Western and Northern Europe, Australia, and New Zealand, and are rapidly increasing in areas of Asia and South America in recent years [6]. IBD typically leads to an overstimulation of the immune system by the normal flora, triggering a cascade of inflammatory responses [50]. Additionally, it often relapses, and conventional treatment is inadequate to maintain long‐term disease remission. Therefore, discovering effective and safe alternative treatment options is crucial. Studies have demonstrated that IBD etiology correlates with gut microbiota dysbiosis and abnormal immune responses [51, 52, 53]. Probiotics have shown efficacy in improving the gut microbiota and regulating the immune response. They have minimal few side effects and a high safety profile, effectively preventing or aiding IBD treatment [54, 55]. Evidence is mounting that *L. sakei* has multiple probiotic functions [38]. However, the immunomodulating functions and mechanisms of different isolated sources of this strain have not been proven yet. In this study, we aimed to screen *L. sakei* from different sources and explore the intraspecific differences of *L. sakei* on IBD, establishing a theoretical basis for further research on *L. sakei* with probiotic functions. Both in vivo and in vitro findings indicated differences in the alleviating effect of *L. sakei* from different sources. Notably, acetylcholine and indole‐3‐acetic acid produced from *L. sakei* CCFM1267 metabolism may be responsible for its effectiveness compared with *L. sakei* QJSNT1L10, which has a poor alleviating effect.

A total of 72 strains of *L. sakei* were screened from 120 fermented samples and fecal samples in China, and 16 strains of *L. sakei* from different sources were selected to investigate their immunomodulatory activities in vitro. In recent years, the Caco‐2 cell model induced by DSS has been observed to be closely related to the early inflammatory response in colitis [56]. When a specific DSS concentration is used to stimulate Caco‐2 cells, it causes cell damage, primarily characterized by heightened intestinal permeability due to the tight connection injury of Caco‐2 cells and other inflammatory damages [57, 58]. Therefore, we used this model to reveal that among 16 strains of *L. sakei* from different sources, *L. sakei* QJSSZ1L4, QJSSZ4L10, CCFM1267, and QGZZYRHMT1L6 could effectively protect the activity of Caco‐2 cells after DSS stimulation. NF‐κB and JNK signaling pathways, which are pivotal pathways in IBD, are frequently observed to be overexpressed in macrophages and intestinal epithelial cells in IBD patients [59], and also trigger a proinflammatory cascade response that promotes the secretion of various proinflammatory cytokines, including IL‐1β, TNF‐α, and IL‐6. Therefore, blocking NF‐κB and JNK signaling pathways is considered a therapeutic modality for treating IBD. We found that *L. sakei* facilitated the immunomodulation of Caco‐2 cells by suppressing the NF‐κB signaling. Simultaneously, *L. sakei* could upregulate the expression of TJ‐related genes in Caco‐2 cells and protect intestinal barrier function. In contrast, *L. sakei* QJSNT1L10 and QGZZYRHMT2L6 lacked the above regulatory effects.

Further validation using animal models revealed that the six strains of *L. sakei* had various degrees of alleviating effects on the disease process in mice with colitis. Among them, *L. sakei* CCFM1267 can effectively restore colon length and mitigate pathological injury in colitis mice. In addition, an intact intestinal barrier is essential for the physiological function of the organism, and for disease prevention [60], and epithelial TJs are important mediators for maintaining the intestinal barrier [61]. Studies have revealed that when the intestinal barrier is damaged, intestinal lumen contents, pathogenic bacteria and symbiotic bacteria will be transferred to the intestinal lamina propria, causing inflammation [62]. Li et al. [63] demonstrated that tree tongue polysaccharide could ameliorate the level of ZO‐1, Occludin, and Claudin‐3 and restore the intestinal barrier, thus effectively relieving DSS‐induced colitis. We also observed that *L. sakei* CCFM1267 mitigates colitis by upregulating the TJ proteins expression and downregulating the levels of proinflammatory cytokines and related inflammatory enzymes, while *L. sakei* QJSNT1L10 had the least alleviating effect. It has been suggested that the occurrence of IBD may be associated with the defective metabolism of SCFAs [64]. Decrease in specific SCFA‐producing bacteria, such as *Faecalibacterium*, in the intestines of colitis patients causes a reduction in the intestinal SCFAs content. This decrease limits the energy supply to the colonic epithelium and diminishes the local regulation of the mucosal inflammatory response [65, 66]. SCFAs effectively maintain intestinal and immune homeostasis, and suppress inflammation primarily by restraining proinflammatory cytokine activity in the intestinal epithelium and inhibiting NF‐κB signaling activation in macrophages [67]. In addition, SCFAs exert inhibitory effects on the proliferation of pathogenic microorganisms like *Escherichia coli* and *Salmonella* within the intestinal environment, competing with them for colonization sites [68]. Shan et al. [69] observed that SCFAs also repair damaged intestinal mucosa due to their ability to stimulate mucus secretion from cupped cells. In light of this, we found that compared with *L. sakei* QJSNT1L10, *L. sakei* CCFM1267 exhibit a more pronounced upregulation of SCFAs, with propionic acid being the most significantly upregulated. It was shown that acetic acid and propionic acid activate GRP43 [70], which in turn effectively reduces inflammation and enhances the mucosal immune response by inhibiting the release of proinflammatory cytokines and promoting the synthesis of anti‐inflammatory cytokines [71]. These findings further explain why *L. sakei* CCFM1267 is more effective than *L. sakei* QJSNT1L10 in relieving colitis. Additionally, *L. sakei* CCFM1267 may better improve some SCFAs‐producing genera, such as *Enterorhabdus*, *Roseburia*, and *Alloprevotella*, which can produce SCFAs with anti‐inflammatory properties like acetic acid and butyric acid to alleviate inflammation [72, 73, 74]. This may also be why the *L. sakei* CCFM1267 group produces more SCFAs than the *L. sakei* QJSNT1L10 group. Also, *L. sakei* CCFM1267 significantly upregulated the abundance of *Adlercreutzia*, a genus known for exerting anti‐inflammatory effects through isoflavone metabolism [47].

Probiotic functionality is intricately linked to their genome and metabolome. Consequently, our study aimed to elucidate the underlying mechanisms through comparative analysis of the strains' genomics and metabolomic profiles (Figure 4A). Numerous untargeted investigations have consistently highlighted substantial perturbations within the gut metabolome in IBD, corroborating the recognized dysbiosis present in gut microbial communities [75]. Particular, specific metabolites of the gut microbiota, namely, SCFAs, bile acids, and tryptophan catabolites, undergo considerable perturbations during the progression of IBD [76]. Among these, microbial tryptophan catabolites, including indole, indoleacrylic acid, indoleacetic acid, and tryptamine, hold considerable therapeutic target potential in treating patients with IBD. These catabolic products have been documented to function as ligands for the aryl hydrocarbon receptor, subsequently leading to modifications in both innate and adaptive immune responses and the preservation of barrier function [77, 78, 79]. On the basis of comparative genomics and metabolomics, the potential mechanisms underlying the differences in the effects of differential strains in alleviating colitis were investigated. ANI and phylogeny revealed that *L. sakei* CCFM1267 and *L. sakei* QJSNT1L10 belong to two subspecies: *L. sakei* subsp. *sakei* and *L. sakei* subsp. *carnosus*, and the differences in their evolutionary relationships may cause variations in their function‐related genes. COGs, KEGG, and carbohydrate annotation analysis revealed that the differential strains significantly differed in carbohydrate utilization‐related genes, such as the GH family, which may be responsible for the differences in SCFAs and thus the different relief effects. Therefore, metabolomics was used to analyze the metabolites of both strains further. Metabolomics analysis of bacterial cell‐free supernatants revealed that both *L. sakei* strains significantly differed in metabolites, with acetylcholine and indole‐3‐acetic acid being the key substances. Acetylcholine triggers the alpha7‐nicotinic acetylcholine receptor‐dependent nicotinic anti‐inflammatory pathway, promoting the production of anti‐inflammatory cytokines for effective relief of colitis [80]. Indole‐3‐acetic acid can be recognized by aromatic hydrocarbon receptors [81], thereby increasing IL‐22 secretion, protecting intestinal epithelial barrier function, and effectively alleviating inflammation [82]. Shi et al. [83] revealed that indole‐3‐acetic acid, a tryptophan metabolite derived from *Lactobacillus plantarum* KLDS 1.0386, effectively alleviated IBD by modulating the IL‐22/STAT3 signaling pathway. This finding is consistent with the results of our study, which suggested that acetylcholine and indole‐3‐acetic acid may be the key metabolites causing the difference in the alleviating effect of different strains of colitis. In this study, the mechanism of the alleviating effect of the differential strains was preliminarily investigated in vitro, and the differences in the metabolites of the differential strains in vitro were analyzed. However, further investigation is necessary to elucidate the alleviating effect of the differential metabolites in the disease, the underlying mechanisms, and whether the differential strains act in vivo by metabolizing the substance.

In summary, IBD is a very complex disease whose direct causes and pathological mechanisms are not fully understood, and its incidence is rising in Asia [84]. Conventional drug therapy has many side effects, however, using probiotics appears to be a promising therapeutic strategy as probiotics have an anti‐inflammatory and gut microbiota regulating effect [85, 86]. Probiotics are well tolerated and have few adverse effects, making them a better alternative for treating IBD. Probiotics can affect various aspects of IBD pathologic etiology and can play a protective role for patients. It is necessary to understand their mechanism of action and all their properties. We found that *L. sakei* isolated from different samples can affect immune cytokine expression, microbiome, and metabolic functions. Different sources of *L. sakei* have varying efficacy in alleviating DSS‐induced colitis. *L. sakei* CCFM1267 emerges as a promising novel probiotic candidate for the treatment of IBD. However, not all probiotics are effective in treating IBD, and several issues need to be resolved, such as determining the dose of probiotics, intervals, and overall treatment duration, which lack unified consensus. The therapeutic efficacy of probiotics in UC and CD and their adverse effects remain controversial due to the lack of available data.

In recent years, there has been a discernible differentiation of bacterial components and metabolites, culminating in the introduction of the term “postbiotic.” This concept holds promise in terms of offering safer options for patient consumption. Emerging evidence suggests that the biological impacts of postbiotics on host well‐being could be on par with those associated with probiotics. This is particularly evident in managing gastrointestinal disorders, even in the absence of live microorganisms [87]. In specific cases, the use of postbiological agents appears more rational. Therefore, the fundamental basis of probiotic relief of colitis can be explored in depth in future studies and further investigated through more rigorous randomized, double‐blind, placebo clinical trials.

## CONCLUSION

In conclusion, we employed both in vitro and in vivo experiments to reveal varying degrees of effectiveness in alleviating symptoms associated with IBD. *L. sakei* CCFM1276, the most effective strain, and *L. sakei* QJSNT1L10, the ineffective strain, were selected to explore further the mechanisms underlying their differential mitigating effects. The findings from the comparative genomics analysis revealed significant variations between the two strains, particularly in genes related to carbohydrate‐active enzymes, such as the GH family. These differences may account for the diverse profiles of SCFAs observed in vivo, thereby causing the distinct therapeutic effects of the two strains. Additionally, our metabolome analysis identified acetylcholine and indole‐3‐acetic acid as the main different metabolites of the two strains.

## METHODS

### Screening of *L. sakei*

*L. sakei*, obtained from fecal samples (JNU20220901IRB01), fermented meat products and fermented vegetable products from several areas of China, were cultured in MRS medium for 24 h at 37°C before being collected by centrifugation at 4°C and 6000*g* for 5 min. Afterward, *L. sakei* was washed and suspended in 0.9% saline solution, and the final concentration was adjusted to 5 × 10^9^ CFU/mL for subsequent animal experiments [88, 89]. All strains identified as *L. sakei* via 16S ribosomal RNA sequencing were stored in 30% glycerol in a −80°C refrigerator [90].

### MTT assay

The culture procedures of Caco‐2 cells obtained from the ATCC Biological Standards Resource Center in the United States were executed in accordance with a previous study [91]. At 85% plate spread, the cells were digested with trypsin containing 0.02% ethylenediaminetetraacetic acid at 37°C and passaged. The cells were seeded in 96‐well plates at a concentration of 6000 cells/well, incubated for 24 h, and washed twice with phosphate‐buffered saline, followed by the addition of 1%, 1.5%, 2%, 2.5%, and 3% DSS, and *L. sakei* at 10^3^−10^7^ CFU/mL, respectively, to determine cell activity 24 h postincubation with the cells by 3‐(4,5)‐dimethylthiahiazo (‐z‐y1)‐3,5‐di‐phenytetrazoliumromide (MTT) assay [92]. The plates were spread as indicated above after selecting the proper molding concentration. A blank medium was added to the control group, and a medium containing 3% DSS was added to the remaining groups. Additionally, bacterial suspensions of different *L. sakei* were added to the experimental groups and coincubated with the cells for 24 h before cell activity was determined.

### RNA extraction and quantitative real‐time polymerase chain reaction (RT‐qPCR)

Total RNA was extracted using the Total RNA Isolation Kit (RC101‐01, 50 rxn) and subsequently reverse‐transcribed into cDNA using the HiScript III Reverse Transcriptase (R302‐01, 1000 U). The ensuing step involved conducting RT‐qPCR on a BioRad‐CFX384 machine (Bio‐Rad), employing SYBR Green Supermix. The PCR reaction was conducted in a total volume of 10 μL. The thermal cycling protocol encompassed an initial denaturation step at 95°C for 30 s, succeeded by 40 cycles of denaturation at 95°C for 5 s, and annealing/extension at 60°C for 30 s. Following the PCR amplification, a melting curve analysis (ranging from 65°C to 95°C, incremented by 0.5°C) was performed to validate the specificity of the amplified fragment. Table S1 lists the sequence of the relevant primers. The relative level of change in target genes was calculated by $2^{-∆∆C_{T}}$.

### Animal protocols

Animal experiments were conducted in a specific‐pathogen‐free (SPF) level barrier environment. In total, 72 male SPF C57BL/6 N mice were randomized into eight groups of nine mice each: control, DSS, *L. sakei* QJSSZ1L4, *L. sakei* QJSNT1L10, *L. sakei* QJSSZ4L10, *L. sakei* CCFM1267, *L. sakei* QGZZYRHMT1L6, and *L. sakei* QGZZYRHMT2L6 groups. All study protocols were approved by the Ethics Committee of Jiangnan University, China (JN. No. 20210615c1040810[176]). A healthy diet and ad libitum access to water were provided for the first week of the experiment for acclimatization. At experiment initialization, the control and DSS groups were gavaged with 0.9% saline solution at 200 μL each, whereas the remaining six groups were gavaged with 200 μL of 5 × 10^9^ CFU/mL of different *L. sakei* suspensions for 1 week, during which time the control group were given normal water, whereas the DSS group and *L. sakei* groups were supplemented with 2.5% DSS. Finally, the mice were killed under isoflurane, and samples of intestinal contents, intestine, and serum were collected.

### Histological evaluation

After fixing the colon samples (approximately 0.5 cm) in paraformaldehyde fixative (4%) for 24 h, the slices were stained with hematoxylin and eosin using the method by Liu et al. [93]. The sections were scanned (Panoramic MI1I digital section scanner) to observe the colonic histopathological damage.

### Determination of TJ, cytokines, and inflammatory enzymes in mouse colon tissue

A 0.5 cm piece of colon tissue was ground in 450 μL of sterile saline and centrifuged for 10 min to collect the supernatant. The contents of TJ and inflammatory enzymes COX‐2, MPO, and iNOS in the supernatant were determined by referring to the relevant operating instructions of the kit from SenBeiJia Biological Technology Co., Ltd. The levels of each inflammatory cytokine in the supernatant were determined using the enzyme immunoassay kits from R&D.

### Determination of gut microbiota composition in mice

Total DNA was extracted from mouse feces using the Fast DNA Spin Kit for Feces, and the V3‐V4 region was amplified using the amplification primers and amplification conditions of Mao et al. [94]. The amplified DNA gels were recovered and purified using the TIANgel Mini Purification Kit, and the DNA was sequenced and analyzed using the Illumina MiSeq PE300 platform.

### Determination of fecal SCFAs in mice

Mouse feces were lyophilized and weighed. Subsequently, 1 mL of saturated sodium chloride solution was added to the sample, which was ground to extract SCFAs according to the method of Tian et al. [95] and analyzed by gaschromatography–mass spectrometry.

### *L. sakei* genome sequencing, assembly, and annotation

The genome of *L. sakei* was sequenced using Illumina Hiseq X Ten, and SOAPdenovo 2 [96] (http://soap.genomics.org.cn/) was used for local assembly and optimization of sequences. The coding sequences in the *L. sakei* genome were predicted using Glimmer [97] (http://ccb.jhu.edu/software/glimmer/index.shtml) software.

### Genome analysis of *L. sakei*

#### Homologous genes and phylogenetic tree analysis

The direct homologous genes and protein sequences of *L. sakei* were obtained via cluster analysis using OrthoMCL v2.0.9 [98]. MAFFT v.−7.313 was used to construct a phylogenetic tree.

#### ANI analysis

Similarities between the genomes of any two *L. sakei* strains were calculated using JSpeciesWS (http://jspecies.ribohost.com/jspeciesws/) and R was used to draw the heat map of the analyses.

#### COG functional gene annotation analysis

The genome sequences of *L. sakei* were aligned using the COGs of proteins database to obtain relevant functional gene classification results.

#### KEGG functional gene annotation

Diamond software was used to annotate the strains in the KEGG database, which provides KEGG Orthology information and compare them with that of the KEGG database to obtain various annotation information, such as gene names and the metabolic pathways involved.

#### Carbohydrate‐active enzyme analysis

The carbohydrate‐activating enzyme‐related genes in the *L. sakei* genome were annotated via the CAZy (http://www.cazy.org/) web page.

### Metabolomics analysis

After two generations of supernatant activation, the supernatant was collected by centrifugation at 6000*g* for 5 min at 4°C after incubation in the exponential phase. The samples were mixed with methanol at a 1:4 ratio, sonicated for 5 min, and left at −20°C for 1 h to remove proteins. The supernatant was spin evaporated after a 15 min centrifugation at 12,000 rpm at 4°C. Subsequently, the precooled methanol–water (methanol to ultrapure water volume ratio of 4:1) was used for resolution. Following repeated centrifugation under the same conditions, a sufficient volume of supernatant was acquired for LC‐MS/MS analysis [99]. Initial screening and derivation of sample metabolite results were performed using Compound Discoverer. SIMCA 14.1 was used to determine the peak areas of the metabolites in the integrated, normalized cationic and anionic modes, as well as the VIP values of the metabolites. OPLS‐DA plots were generated to analyze the clustering results of the samples.

### Statistical analysis

All experimental results in this study were the data of three parallel measurements. Microsoft Excel, GraphPad 8, and R were used to sort and map the experimental data statistically. All the data are expressed as the mean ± standard error of the mean. A one‐way analysis of variance was used to analyze the results and determine statistical significance. *p* < 0.05 was considered statistically significant.

## AUTHOR CONTRIBUTIONS

**Yaru Liu**: Data curation, data analysis, writing—original draft, and writing—review and editing. **Hui Duan**: Investigation, writing—original draft, and writing—review and editing. **Ying Chen**: Methodology, validation, data curation, and data analysis. **Chengcheng Zhang**: Conceptualization and data curation. **Qixiao Zhai**: Conceptualization and funding acquisition. **Leilei Yu**: Conceptualization, funding acquisition, supervision, and writing—review and editing. **Jianxin Zhao, Fengwei Tian, and Wei Chen**: Conceptualization and funding acquisition. **Arjan Narbad**: Conceptualization. All authors have read the final manuscript and approved it for publication.

## CONFLICT OF INTEREST STATEMENT

The authors have declared no competing interests.

## ETHICS STATEMENT

The ethics application (JNU20220901IRB01) was approved by the Medical Ethics Committee Jiangnan University. The ethics application (JN. No. 20210615c1040810[176]) was approved by the Ethics Committee of Jiangnan University.

## Supporting information

Supporting information.

Supporting information.

## Data Availability

Research data are not shared. Data generated in this manuscript is included in Supporting Information files. Supporting Information materials (figures, tables, scripts, graphical abstract, slides, videos, Chinese translated version and update materials) may be found in the online DOI or iMeta Science http://www.imeta.science/.
