# Supplementary material for: Intraspecific difference of Latilactobacillus sakei in inflammatory bowel diseases: Insights into potential mechanisms through comparative genomics and metabolomics analyses
Source: Imeta. 2023 Sep 25;2(4):e136. doi: 10.1002/imt2.136 (PMC10989848; doi:10.1002/imt2.136)
Supplement: Supplementary file 1 — Supporting information. [file IMT2-2-e136-s001.docx]

Supporting information for:

**Intra-specific difference of *Latilactobacillus sakei* on inflammatory bowel diseases: insights into potential mechanisms through comparative genomics and metabolomics analyses**

**Running title**: Intra-specific difference of *Latilactobacillus sakei* on inflammatory bowel diseases

Yaru Liu^1,2#^, Hui Duan^1,2#^, Ying Chen^1,2^, Chengcheng Zhang^1,2^, Jianxin Zhao^1,2,3,4^, Arjan Narbad^4,5^, Fengwei Tian^1,2,4^, Qixiao Zhai^1,2,4^, Leilei Yu^1,2,4*^, Wei Chen^1,2,3,4^

^1^ State Key Laboratory of Food Science and Resources, Jiangnan University, Wuxi, 214122, China

^2^ School of Food Science and Technology, Jiangnan University, Wuxi, Jiangsu, 214122, China

^3^ National Engineering Research Center for Functional Food, Jiangnan University, Wuxi, Jiangsu, 214122, China

^4^ International Joint Research Laboratory for Probiotics at Jiangnan University, Wuxi, Jiangsu, 214122, China

^5^ Gut Health and Microbiome Institute Strategic Programme, Quadram Institute Bioscience, Norwich, 16 NR4 7UQ, UK

^#^ These authors contributed equally: Yaru Liu, Hui Duan

*Corresponding author: edyulei@126.com (Leilei Yu)

**Figure S1.** **Effect of different DSS concentration on Caco-2 cell viability.** Different superscript lowercase letters (a-c) in the graph indicate significant differences between groups (*p* < 0.05) within the row by Tukey's multiple comparisons test (n=6).

**Figure S2. Effect of different concentration of *L. sakei* on viability of Caco-2 cells.** Different superscript lowercase letters (a-c) in the graph indicate significant differences between groups (*p* < 0.05) within the row by Tukey's multiple comparisons test (n=6).

**Figure S3.** **Effects of *L. sakei* on colon length in colitis mice.** *L. sakei* QJSSZ4L10 and CCFM1267 could improve abnormal colon length, and the other four strains had no protective effect on colon length.

**
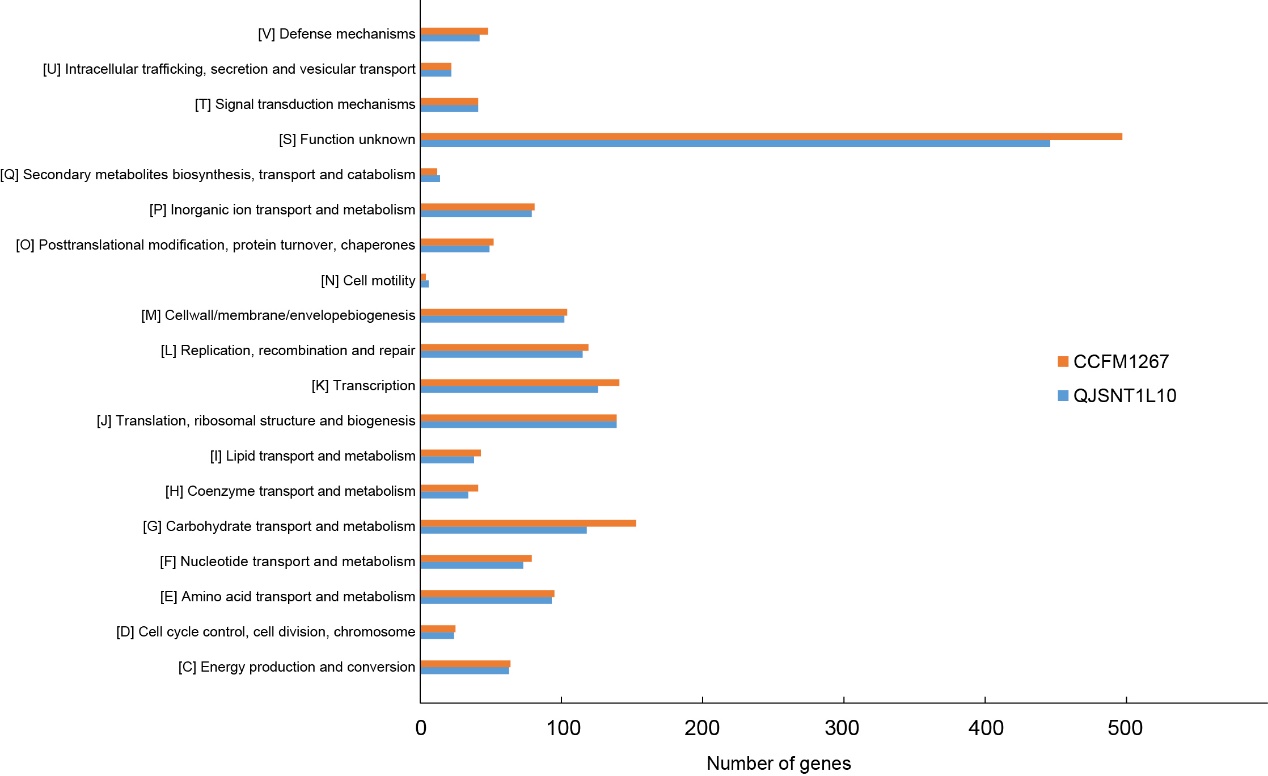
**

**Figure S4.** **COG functional gene composition of *L. sakei*.** The functional genes of the core genome of the two strains were distributed in 19 functions, and did not differ significantly. However, compared with *L. sakei* QJSNT1L10, *L. sakei* CCFM1267 had more genes encoding this function in [G] Carbohydrate transport and metabolism.

**Figure S5.** **KEGG database notes of *L. sakei*.** The functional genes of the two strains of *L. sakei* were mainly distributed in 37 functional categories. In addition to global and overview maps, functional genes are mainly distributed in five functions: carbohydrate metabolism, nucleotide metabolism, membrane transport, translation, replication and repair. According to the gene distribution of the two strains of *L. sakei*, compared with *L. sakei* QJSNT1L10, the functional genes of *L. sakei* CCFM1267 are more distributed in carbohydrate metabolism.
